# Long‐Term Observed Risk of Clinically Significant Prostate Cancer After Negative mpMRI and Systematic Biopsy in Biopsy‐Naïve Men: A MULTIPROS Subanalysis

**DOI:** 10.1155/proc/9470087

**Published:** 2026-08-20

**Authors:** Wafa D. Aloufi, Xinyu Zhang, Magdalena Szewczyk-Bieda, Ghulam Nabi, Cheng Wei, Luigi Manfredi

**Affiliations:** ^1^ University of Dundee School of Medicine, Dundee, UK; ^2^ Taif University, Taif, Makkah Province, Saudi Arabia, tu.edu.sa; ^3^ Division of Population Health and Genomics, University of Dundee, Dundee, UK, dundee.ac.uk; ^4^ Ninewells Hospital and Medical School, Dundee, UK, dundee.ac.uk

**Keywords:** active surveillance, biopsy, detection of cancer, magnetic resonance imaging, prostate-specific antigen, prostatic neoplasms, TRUS

## Abstract

**Background:**

mpMRI is now widely used before prostate biopsy and has improved the detection of clinically significant prostate cancer (csPCa). However, the subsequent risk of csPCa among men with both negative mpMRI and negative systematic biopsy (SBx) remains uncertain.

**Purpose:**

To evaluate the observed medium‐term risk of csPCa in biopsy‐naïve men with initially negative mpMRI and negative SBx.

**Methods:**

This post hoc longitudinal follow‐up subanalysis included biopsy‐naïve men from the prospective multicenter MULTIPROS trial who had negative baseline mpMRI (PI‐RADS ≤ 2) and negative SBx. Of the 582 enrolled men, 169 had negative mpMRI; 138 underwent baseline SBx, of whom 8 had csPCa, and 31 had < 12 months of follow‐up, leaving 99 men for analysis. Follow‐up was PSA‐based, with repeat mpMRI and biopsy performed when clinically indicated. Kaplan–Meier analysis estimated csPCa‐free survival.

**Results:**

During a mean follow‐up of 61.8 ± 22.1 months, four patients were diagnosed with csPCa (4/99; 4.0%). The mean time from initial negative mpMRI to csPCa diagnosis was 68.3 ± 22.4 months. Repeat biopsy was performed in 16 patients. csPCa‐free survival was 100% at 24 months and 97.3% at 60 months (95% CI: 93.7%–100%).

**Conclusion:**

In this selected cohort of biopsy‐naïve men with both negative mpMRI and negative SBx, the observed incidence of csPCa during medium‐term follow‐up was low but based on only four events. Because repeat imaging and biopsy were selectively triggered rather than systematically performed, the true incidence of csPCa may be underestimated. Continued PSA‐based monitoring, with repeat mpMRI and biopsy when clinically indicated, remains important.

## 1. Introduction

Prostate cancer (PCa) remains one of the most common malignancies in men worldwide, with early and accurate detection playing a crucial role in improving patient outcomes. For over four decades, systematic biopsy (SBx) has been the standard diagnostic approach [1]. However, SBx has notable limitations, including the risk of missing clinically significant PCa (csPCa) and the overdiagnosis of insignificant PCa. Given these limitations, mpMRI has emerged as a valuable imaging modality and has markedly advanced the detection and management of csPCa [2–4].

Several studies [5–8] have demonstrated that mpMRI enhances the detection and localization of csPCa, shifting the diagnostic paradigm toward imaging before biopsy. Some studies demonstrated that combining MRI‐targeted biopsy (MRI‐TBx) with SBx increases csPCa detection compared to SBx alone [9–13], while others reported that MRI‐TBx alone can outperform SBx in identifying csPCa [14–16]. Current guidelines recommend mpMRI as the first‐line diagnostic tool for patients with suspected PCa, enabling targeted biopsies when the likelihood of csPCa is intermediate to very high. This approach improves csPCa detection, reduces the diagnosis of insignificant PCa, minimizes unnecessary random biopsies, and enhances overall risk assessment. Recent meta‐analyses [17] support combined MRI‐TBx plus random biopsy as superior to SBx alone for csPCa detection. Conversely, when the likelihood of PCa is low, repeat biopsies are often deferred in favor of continued clinical and biochemical monitoring [18, 19]. However, uncertainty remains regarding long‐term outcomes in men with negative mpMRI.

Despite these advantages, the negative predictive value (NPV) of mpMRI varies widely (from 86.8% to 97.1%) depending on study design, cancer prevalence, and the definition of csPCa [19, 20]. In addition, the long‐term false‐negative rate of mpMRI remains uncertain, particularly in men who initially had negative findings. At present, there is no clear consensus on the optimal follow‐up strategy for patients with negative mpMRI but persistent clinical suspicion of PCa, making decisions highly dependent on shared clinical judgment. Current risk‐adapted strategies may also consider PSA density, PSA kinetics, DRE findings, family history, and secondary biomarkers when deciding whether repeat imaging or biopsy is required after negative mpMRI.

Recently, the results of the MULTIPROS trial [14] were published. The primary aim of the trial was to evaluate the diagnostic accuracy of prebiopsy‐positive mpMRI in detecting csPCa, using radical prostatectomy (RP) specimens as the reference standard. In addition, the trial compared the performance of combined ultrasound (US) and MRI fusion–targeted biopsy with SBx in biopsy‐naïve patients. However, a critical question remains: what are the follow‐up outcomes for men with negative mpMRI and SBx?

This study aimed to evaluate the observed long‐term incidence of csPCa after initially negative mpMRI and negative SBx in biopsy‐naïve men from the MULTIPROS cohort. We also describe the follow‐up pathway leading to diagnosis, including PSA monitoring, repeat mpMRI, and repeat biopsy. Given the selective nature of repeat investigations, the findings are interpreted as observed outcomes within a PSA‐triggered clinical follow‐up pathway rather than as protocol‐mandated re‐biopsy outcomes.

## 2. Materials and Methods

### 2.1. Study Design and Setting

The MULTIPROS trial was a prospective, multicenter, randomized diagnostic accuracy study assessing prebiopsy mpMRI and image‐guided biopsy in biopsy‐naïve men with suspected PCa [14, 21]. The parent trial recruited men aged 40–75 years with PSA < 20 ng/mL and DRE findings of pT2b or less between February 2015 and August 2020. The present study was a post hoc retrospective longitudinal follow‐up cohort subanalysis of men with negative baseline mpMRI, defined as PI‐RADS ≤ 2, and negative baseline SBx. Of 582 biopsy‐naïve men enrolled in MULTIPROS, 169 had negative baseline mpMRI. According to the MULTIPROS pathway, men with negative mpMRI were intended to undergo standard‐care SBx. However, 31 of 169 men did not undergo SBx, mainly because of COVID‐19‐related service disruption, and were therefore not eligible for the present double‐negative cohort. Among the remaining 138 men who underwent SBx, 8 were diagnosed with csPCa at baseline and were excluded. A further 31 patients were excluded because they had < 12 months of available follow‐up. The final cohort therefore included 99 men with both negative baseline mpMRI and negative baseline SBx.

### 2.2. Multiparametric MRI and Biopsy Protocol

All participants underwent prebiopsy mpMRI. The MRI systems utilized across institutions included a 3‐T Magnetom Trio‐PrismaFIT (Siemens; NHS Tayside), a 3‐T Achieva (Philips; NHS Grampian), and a 1.5‐T Ingenia (Philips; Royal Free London). The imaging protocols, standardized according to the European Society of Urogenital Radiology (ESUR) guidelines [22], comprised T2‐weighted imaging, diffusion‐weighted imaging (DWI) with high b‐value, and dynamic contrast–enhanced (DCE) sequences. A three‐dimensional T2‐weighted fast spin‐echo sequence was additionally acquired for biopsy planning [14]. All mpMRI scans were prospectively reviewed by experienced uroradiologists, each with over five years of expertise and an annual caseload exceeding 200 mpMRI scans. The radiologists were blinded to clinical information at reporting (including age, PSA level, and DRE findings) and evaluated MRI using PI‐RADS V2.0 [23].

Men with negative mpMRI underwent standard‐care 12‐core TRUS‐guided SBx. In the MULTIPROS pathway, biopsy was generally performed within 1 week of the mpMRI examination. During follow‐up, patients who developed a positive repeat mpMRI underwent TRUS‐guided SBx combined with targeted biopsy, with approximately 10–12 cores obtained according to clinical practice.

### 2.3. Follow‐Up and Inclusion/Exclusion Criteria

Eligible patients were men from the MULTIPROS trial with negative baseline mpMRI (PI‐RADS ≤ 2), negative baseline SBx, and at least 12 months of available follow‐up. Patients were excluded if they did not undergo baseline SBx, had csPCa detected on baseline SBx, or had < 12 months of follow‐up.

Follow‐up was based on routine clinical and biochemical monitoring. According to the local prebiopsy MRI pathway, patients with PI‐RADS 1–2 negative MRI were advised to undergo repeat PSA testing at 3 months. Repeat investigation was considered when PSA was elevated on two occasions in the absence of urinary tract infection, or when PSA elevation was accompanied by abnormal DRE. PSA elevation was defined according to contemporaneous local age‐adjusted clinical reference ranges, rather than by applying a single universal PSA threshold. No fixed PSA velocity threshold was specified, and although PSA density was calculated for analysis, it was not documented as a prespecified trigger for repeat mpMRI or biopsy. Patients without repeat biopsy were censored at their last available PSA follow‐up. Patients diagnosed with csPCa during follow‐up were recorded as events. All csPCa diagnoses in this cohort occurred after repeat mpMRI prompted by PSA elevation and/or persistent clinical suspicion.

### 2.4. Statistical Analysis

Patient demographics and clinical characteristics, including age, PSA levels, prostate volume (mL), and PSA density (ng/mL^2^), were collected and summarized using descriptive statistics. Baseline characteristics of the 31 patients excluded because of < 12 months of follow‐up were also summarized descriptively and compared with the included cohort to assess possible attrition bias. Continuous variables were tested for normality using the Kolmogorov–Smirnov test. Variables that were normally distributed are presented as mean ± standard deviations (SDs), while non‐normally distributed variables are reported as medians with interquartile ranges (IQRs). Categorical variables, such as DRE findings, PI‐RADS, the number of biopsies, and the number of mpMRI scans per patient, are described using frequencies and percentages. The primary outcome was the detection of csPCa, defined as a Gleason score of ≥ 3 + 4. Because only four csPCa events occurred, comparisons between patients with and without csPCa were presented descriptively only, and no inferential between‐group testing was performed for patients, as shown in Table 1. No multivariable modeling was performed due to the low number of events. Kaplan–Meier analysis was used to estimate csPCa‐free survival, with 95% confidence intervals reported where estimable. All analyses were performed using SPSS Version 29.0 (SPSS Inc., IBM Corp., Armonk, NY, USA).

**TABLE 1:** Patients’ characteristics and outcomes.

Variables	Total cohort	csPCa	No csPCa
Number of patients (%)	99 (100%)	4 (4%)	95 (96%)
Baseline PSA level (ng/mL), median (IQR)	7.2 (5.7–8.3)	6.7 (4.8–7.5)	7.2 (5.7–8.3)
Baseline DRE results:	—	—	—
Abnormal DRE, *n* (%)	29 (29.3%)	0 (0%)	29 (30.5%)
Normal DRE, *n* (%)	70 (70.7%)	4 (100%)	66 (69.5%)
Follow‐up variables	—	—	—
Age (years), median (IQR)	71 (64–75)	72 (67.3–76.7)	70 (64–75)
PSA level (ng/mL), median (IQR)	7.7 (6.2–10.5)	12 (9.7–16.5)	7.7 (6.2–10.1)
Prostate volume (mL), mean ± SD	83.5 ± 34.3	84.5 ± 17.4	83.5 ± 34.9
Prostate density (ng/mL^2^), median (IQR)	0.10 (0.07–0.14)	0.14 (0.12–0.22)	0.10 (0.07–0.14)
Follow‐up period (months), mean ± SD	61.8 ± 22.1	68.3 ± 22.4	61.1 ± 21.9
Number of biopsy procedures/patient	—	—	—
< 2 Bx (%)	83 (83.8%)	0 (0%)	83 (87.4%)
≥ 2 Bx (%)	16 (16.2%)	4 (100%)	12 (12.6%)
Number of mpMRI scans/patient:	—	—	—
< 2 mpMRI (%)	57 (57.6%)	0 (0%)	57 (60%)
≥ 2 mpMRI (%)	42 (42.4%)	4 (100%)	38 (40%)

*Note:* Values are presented as mean ± SD, median (IQR), or *n* (%), as appropriate. Because only four csPCa events occurred, no inferential between‐group testing was performed; comparisons are descriptive only.

## 3. Results

### 3.1. Cohort Derivation and Baseline Characteristics

**FIGURE 1:**
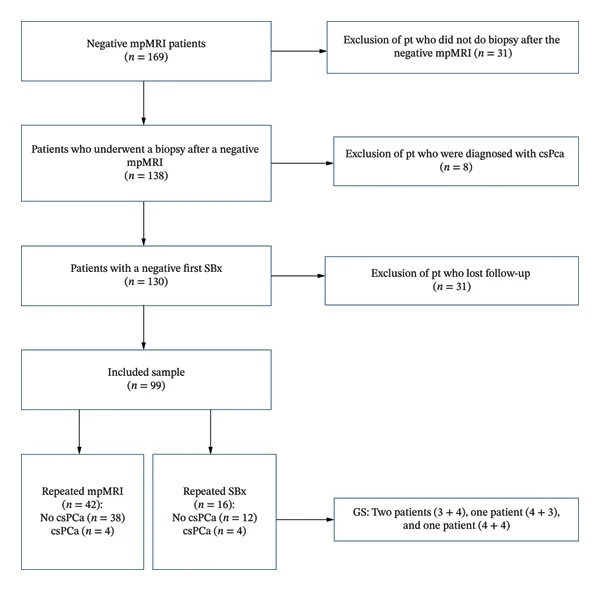
Study design and participant flowchart.

Figure 1 illustrates the cohort derivation. Of 582 biopsy‐naïve men in MULTIPROS, 169 had negative baseline mpMRI. After excluding 31 men who did not undergo baseline SBx, eight diagnosed with csPCa on baseline SBx, and 31 with < 12 months of follow‐up, 99 men formed the final double‐negative follow‐up cohort. Baseline characteristics of the short‐follow‐up excluded group were broadly similar to those of the included cohort for available variables (Supporting Table S2). Their age, baseline PSA, prostate volume, and PSA density were broadly similar to those of the included cohort.

In this cohort, the median (IQR) age was 71 (64–75) years, and the median (IQR) serum PSA level was 7.7 (6.2–10.5) ng/mL. The mean ± SD of the prostate volume was 83.5 ± 34.3 mL. The median PSA value was based on the most recent PSA level for patients without csPCa and the last PSA measurement before diagnosis for those who developed csPCa. Baseline DRE was abnormal in 29 patients. During follow‐up, 42 patients underwent repeat mpMRI, and 16 patients underwent repeat biopsy. Therefore, the observed csPCa detection rate reflects a PSA‐triggered clinical follow‐up pathway rather than systematic protocol‐mandated repeat biopsy of the entire cohort. Three patients died during follow‐up, none of whom had PCa‐related causes. Patient characteristics and outcomes are summarized in Table 1.

### 3.2. Descriptive Analysis of csPCa Cases

During follow‐up, 4 of 99 patients were diagnosed with csPCa. The mean time from initial negative mpMRI to csPCa diagnosis was 68.3 ± 22.4 months. Final Gleason scores were 3 + 4 in two patients, 4 + 3 in one, and 4 + 4 in one. Two patients underwent RP and two received radiotherapy.

Repeat mpMRI demonstrated newly emergent suspicious lesions classified as PI‐RADS 4 (*n* = 3) or PI‐RADS 5 (*n* = 1). All lesions were located in the peripheral zone and measured 7–24 mm in maximum diameter. Each showed diffusion restriction with corresponding T2 signal abnormality and dynamic contrast enhancement where performed. Capsular contact was observed in three cases, without definite extracapsular extension. Biopsy findings were concordant with imaging in all cases, and PSA elevation preceded repeat imaging and biopsy. Repeat biopsy in these four patients consisted of TRUS‐guided SBx combined with targeted biopsy after positive repeat mpMRI (Table 2).

**TABLE 2:** Clinical details for positive patients.

Patients	Repeat mpMRI PI‐RADS	No. of mpMRI scans	No. of repeat biopsy procedures	PSA at diagnosis (ng/mL)	Gleason score	Index lesion location and size	Location of positive cores	Time interval (months)
No. 1	4	3	2	11.4	4 + 4	Left peripheral zone at mid‐gland, 8 mm	‐Left mid lateral	48

No. 2	4	4	3	17.8	4 + 3	Left peripheral zone at mid‐gland, 10 mm	‐Left upper lateral	88
							‐Left base	
							‐Left mid lateral	
							‐Left paramidline	
							‐Left lower lateral	

No. 3	4	4	2	9.1	3 + 4	Right posterior peripheral zone at the apex, 7 mm	‐Right mid lateral	87
							‐Right paramidline	
							‐Right lower lateral	
							‐Left base	
							‐Left mid lateral	
							‐Left paramidline	

No. 4	5	3	3	12.6	3 + 4	Left peripheral zone at mid‐gland, 24 mm	‐Left upper lateral	50
							‐Left lateral mid gland	
							‐Left lateral apex	

### 3.3. csPCa‐Free Survival During Follow‐Up

**FIGURE 2:**
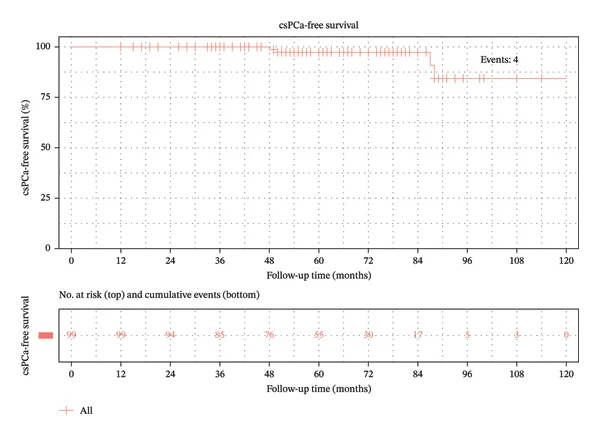
csPCa diagnosis‐free survival curve for patients with initially negative mpMRI and negative SBx.

Kaplan–Meier analysis showed csPCa‐free survival of 100% at 24 months and 97.3% at 60 months (95% CI: 93.7%–100%) (Figure 2). Estimates beyond 60 months should be interpreted cautiously because few patients remained at risk and repeat biopsy was not systematically performed.

## 4. Discussion

In this post hoc follow‐up subanalysis of the MULTIPROS trial, we assessed long‐term outcomes in biopsy‐naïve men with both negative baseline mpMRI (PI‐RADS ≤ 2) and negative SBx. Over a mean follow‐up of 61.8 ± 22.1 months, csPCa was detected in 4 of 99 patients, indicating a low observed incidence in this selected double‐negative cohort. However, interpretation should remain cautious, as repeat biopsy was selectively triggered by PSA elevation and/or clinical suspicion rather than performed systematically.

Selection and attrition bias should be considered. The final cohort excluded 31 men who did not undergo baseline SBx, mainly because of COVID‐19‐related service disruption, and 31 men with < 12 months of follow‐up. Although the short‐follow‐up excluded group appeared broadly comparable for available baseline variables, the analyzed cohort remains a selected subset of the original MULTIPROS population. Therefore, the Kaplan–Meier estimates reflect observed csPCa‐free survival within a PSA‐triggered pathway and may underestimate the true incidence of csPCa.

In the initial MULTIPROS analysis, 8 of 138 men (5.8%) with negative mpMRI were diagnosed with csPCa on baseline SBx [14]. This finding indicates that biopsy omission after negative mpMRI may miss a small but clinically important proportion of csPCa cases, particularly in men with persistent PSA elevation or clinical suspicion.

In the present follow‐up cohort of 99 men with both negative mpMRI and negative SBx, 97.3% remained free from csPCa at 60 months. All subsequent diagnoses were preceded by PSA elevation and/or renewed clinical concern, supporting a risk‐adapted surveillance approach. Our findings align with the 3.0% detection rate reported in the FUTURE trial by Exterkate et al. [24], which followed 431 men with prior negative biopsies and nonsuspicious mpMRI for a median of 41 months. The slightly higher observed rate in our study may partly reflect longer follow‐up duration, although differences in population selection and follow‐up pathways limit direct comparison. Similarly, Panebianco et al. [25] reported a 95% csPCa‐free survival at two years following negative mpMRI, while Venderink et al. [26] observed a 0.4% rate over three years in a mixed cohort. Differences in follow‐up duration, patient selection, and diagnostic pathways likely explain this variability. Two systematic reviews further reinforce the high NPV of mpMRI, with pooled estimates ranging from 86.8% to 97.1% [20, 27]. Nevertheless, variability across studies highlights the importance of risk‐adapted follow‐up strategies rather than uniform biopsy omission. When repeat biopsy is clinically triggered after prior negative assessment, biopsy strategy remains important. In this cohort, positive repeat mpMRI was followed by combined TRUS‐guided systematic and targeted biopsy, aligning with evidence supporting MRI‐directed sampling while acknowledging the added value of systematic cores [28].

A strength of this study is that it represents a secondary analysis of a prospectively recruited trial cohort, with extended follow‐up averaging 60 months. By focusing exclusively on biopsy‐naïve men with both negative mpMRI and SBx, it provides clinically relevant insight into long‐term outcomes in this specific population. Given the median age of 71 years, cancers detected 48–88 months after the initial negative assessment may represent incident disease rather than lesions missed at baseline. Notably, all delayed csPCa cases were identified on repeat mpMRI as newly suspicious PI‐RADS 4–5 lesions within the peripheral zone, with clear imaging–pathology concordance at biopsy. This supports the role of repeat mpMRI in detecting clinically significant interval disease in selected patients with rising PSA following an initially negative assessment.

Several limitations should be acknowledged. The small cohort (*n* = 99) and low number of csPCa events (*n* = 4) limit statistical power and generalizability. Reliance on PSA‐triggered repeat imaging and biopsy may have introduced verification bias, as PSA elevation can reflect benign conditions [29, 30], while stable PSA does not fully exclude csPCa. Attrition bias also remains possible, as 31 patients with less than 12 months of follow‐up were excluded and their outcomes were unavailable, despite broadly similar baseline characteristics for available variables. In addition, 31 men with negative mpMRI did not undergo baseline SBx, mainly due to COVID‐19‐related service disruption, meaning the final cohort may not fully represent all men with negative mpMRI in the parent trial.

Larger prospective studies with protocol‐driven follow‐up are needed to validate these findings and define risk‐adapted surveillance strategies incorporating PSA kinetics, PSA density, DRE findings, biomarkers, and repeat imaging. Future studies should use predefined triggers for repeat mpMRI and biopsy and standardized imaging and biopsy criteria. Blinded re‐review of baseline mpMRI in men later diagnosed with csPCa may help distinguish incident cancers from initial perceptual misses.

## 5. Conclusions

In this selected double‐negative cohort, the observed incidence of csPCa during approximately 5 years of follow‐up was low, with 97.3% csPCa‐free survival at 60 months. However, the small cohort, few events, and PSA‐/suspicion‐triggered follow‐up mean that the true incidence may be underestimated. Continued PSA‐based monitoring, with repeat mpMRI and biopsy when clinically indicated, remains important.

## Author Contributions

Wafa D. Aloufi: methodology, investigation, writing–original draft, review, and editing. Xinyu Zhang: statistical analysis, methodology, review, and editing. Magdalena Szewczyk‐Bieda: methodology, review, and editing. Ghulam Nabi: supervision. Cheng Wei: supervision. Luigi Manfredi: supervision, review, and editing.

## Funding

This research was conducted as part of a PhD scholarship funded by Taif University, Saudi Arabia.

## Disclosure

All authors have read and agreed to the published version of the manuscript. The funder had no role in study design, data collection, analysis, interpretation, manuscript preparation, or the decision to submit for publication.

## Ethics Statement

The original MULTIPROS trial received ethical approval from the East of Scotland Research Ethics Committee 1 (Ref: 14/ES/1070; approved 20 November 2014), and written informed consent was obtained from all participants, including consent for future related research. This retrospective follow‐up subanalysis was conducted under NHS Tayside Caldicott approval: IGTCAL‐2024‐167, granted on 2 September 2024.

## Consent

Please see the Ethics Statement.

## Conflicts of Interest

The authors declare no conflicts of interest.

## Supporting Information

Additional supporting information can be found online in the Supporting Information section.

## Supporting information


**Supporting Information** Supporting 1. Supporting File S1: STROBE checklist for reporting observational studies.

**Supporting Information** Supporting 2. Supporting File S2: Baseline characteristics of included patients and those excluded because of < 12 months of follow‐up.

## Data Availability

The data that support the findings of this study are not publicly available because they contain sensitive patient‐level clinical and imaging information. The data cannot be shared outside the study team under the current ethical and information governance approvals. Any request for access would require review and approval by the relevant sponsoring institution, data controller, and ethics and information governance bodies, in accordance with applicable data protection regulations.
