# Supplementary material for: Long‐Term Observed Risk of Clinically Significant Prostate Cancer After Negative mpMRI and Systematic Biopsy in Biopsy‐Naïve Men: A MULTIPROS Subanalysis
Source: Prostate Cancer. 2026 Aug 20;2026:9470087. doi: 10.1155/proc/9470087 (PMC13492054; doi:10.1155/proc/9470087)
Supplement: Supplementary file 1 [file PROC-2026-9470087-s001.docx]

Supplementary Table S2. Baseline characteristics of included and short-follow-up excluded patients

| Variables | Included cohort n=99 | Excluded <12-month follow-up n=31 |
| --- | --- | --- |
| Age, median (IQR), years | 71 (64–75) | 72 (66–77) |
| Baseline PSA, median (IQR), ng/mL | 7.7 (6.2–10.5) | 6.5 (5.2–9.3) |
| Prostate volume, mean ± SD, mL | 83.5 ± 34.3 | 76.5 ± 39.5 |
| PSA density, median (IQR), ng/mL² | 0.10 (0.07–0.14) | 0.08 (0.06–0.16) |
| Abnormal DRE, n (%) | 29 (29.3%) | 12 (38.7%) |
| Normal DRE, n (%) | 70 (70.7%) | 19 (61.3%) |
| Follow-up duration, mean ± SD, months | 61.8 ± 22.1 | 2.8 ± 2.9 |

^For patients in the excluded group whose recorded last follow-up date preceded baseline mpMRI, the baseline SBx date was used as the last verifiable clinical contact. All 31 excluded patients had <12 months of available follow-up.^
