# Supplementary material for: Long‐Term Observed Risk of Clinically Significant Prostate Cancer After Negative mpMRI and Systematic Biopsy in Biopsy‐Naïve Men: A MULTIPROS Subanalysis
Source: Prostate Cancer. 2026 Aug 20;2026:9470087. doi: 10.1155/proc/9470087 (PMC13492054; doi:10.1155/proc/9470087)
Supplement: Supplementary file 2 [file PROC-2026-9470087-s002.docx]

STROBE Statement—checklist of items that should be included in reports of observational studies

|  | Item No. | Recommendation | Page  No. | Relevant text from manuscript |
| --- | --- | --- | --- | --- |
| **Title and abstract** | 1 | (*a*) Indicate the study’s design with a commonly used term in the title or the abstract | 2 | Abstract Methods: “This post-hoc longitudinal follow-up sub-analysis included biopsy-naïve men from the prospective multicentre MULTIPROS trial…” |
|  |  | (*b*) Provide in the abstract an informative and balanced summary of what was done and what was found | 2 | The structured abstract reports the Background, Purpose, Methods, Results, and Conclusion, including the cohort derivation, follow-up approach, four csPCa events, and 60-month csPCa-free survival. |
| Introduction | | | |  |
| Background/rationale | 2 | Explain the scientific background and rationale for the investigation being reported | 3 | The Introduction explains the limitations of SBx, the role and negative predictive value of mpMRI, uncertainty regarding long-term false-negative outcomes, and the lack of consensus on follow-up after negative mpMRI. |
| Objectives | 3 | State specific objectives, including any prespecified hypotheses | 4 | “This study aimed to evaluate the observed long-term incidence of csPCa after initially negative mpMRI and negative SBx… We also describe the follow-up pathway leading to diagnosis…” |
| Methods | | | |  |
| Study design | 4 | Present key elements of study design early in the paper | 4-5,7 | “The MULTIPROS trial was a prospective, multicentre, randomised controlled study…” and “This paper reports a prospective sub-analysis…” |
| Setting | 5 | Describe the setting, locations, and relevant dates, including periods of recruitment, exposure, follow-up, and data collection | 4 | “Between February 2015 and August 2020…” plus MRI centres and follow-up description. |
| Participants | 6 | (*a*) *Cohort study*—Give the eligibility criteria, and the sources and methods of selection of participants. Describe methods of follow-up  *Case-control study*—Give the eligibility criteria, and the sources and methods of case ascertainment and control selection. Give the rationale for the choice of cases and controls  *Cross-sectional study*—Give the eligibility criteria, and the sources and methods of selection of participants | 4–5 | Eligibility and selection are described in Section 2.1 and Section 2.3. Follow-up included repeat PSA at 3 months, routine clinical/biochemical monitoring, and clinically triggered repeat mpMRI/biopsy. |
|  |  | (*b*) *Cohort study*—For matched studies, give matching criteria and number of exposed and unexposed  *Case-control study*—For matched studies, give matching criteria and the number of controls per case | N/A |  |
| Variables | 7 | Clearly define all outcomes, exposures, predictors, potential confounders, and effect modifiers. Give diagnostic criteria, if applicable | 4–6 | The double-negative cohort is defined by PI-RADS ≤2 and negative baseline SBx. Age, PSA, prostate volume, PSA density, DRE, biopsy/mpMRI counts, and the primary outcome are specified; csPCa is defined as Gleason score ≥3+4. |
| Data sources/ measurement | 8* | For each variable of interest, give sources of data and details of methods of assessment (measurement). Describe comparability of assessment methods if there is more than one group | *4-5,7* | MRI platforms and protocol, radiologist experience/blinding, PI-RADS v2.0, PSA/DRE follow-up, statistical variable definitions. |
| Bias | 9 | Describe any efforts to address potential sources of bias | 2, 5, 7, 10–12 | Attrition was explored by descriptively comparing the 31 patients with <12 months of follow-up with the included cohort (p. 5). Selective repeat investigation and possible underestimation are stated in the Abstract/Results and selection, attrition, and verification bias are discussed with their likely direction in the Discussion. |
| Study size | 10 | Explain how the study size was arrived at | 4, 6–7 | The cohort derivation is reported: 582 enrolled; 169 negative mpMRI; 31 without baseline SBx; 138 underwent SBx; 8 baseline csPCa; 31 with <12 months of follow-up; 99 analysed. Figure 1 presents the flow. |

Continued on next page

| Quantitative variables | 11 | Explain how quantitative variables were handled in the analyses. If applicable, describe which groupings were chosen and why | 4–8 | Continuous variables were tested for normality and summarised as mean ± SD or median (IQR); categorical variables were summarised as n (%). Reported boundaries include PI-RADS ≤2, ≥12 months of follow-up, and <2 versus ≥2 biopsy procedures/mpMRI scans. |
| --- | --- | --- | --- | --- |
| Statistical methods | 12 | (*a*) Describe all statistical methods, including those used to control for confounding | 5–6 | Descriptive statistics, Kolmogorov–Smirnov testing, frequency/percentage summaries, and Kaplan–Meier estimation with 95% confidence intervals are described. No inferential between-group testing or multivariable modelling was performed because only four events occurred. Analyses used SPSS v29.0. |
|  |  | (*b*) Describe any methods used to examine subgroups and interactions | N/A |  |
|  |  | (*c*) Explain how missing data were addressed | N/A |  |
|  |  | (*d*) *Cohort study*—If applicable, explain how loss to follow-up was addressed  *Case-control study*—If applicable, explain how matching of cases and controls was addressed  *Cross-sectional study*—If applicable, describe analytical methods taking account of sampling strategy | 5, 10–12 | Patients without repeat biopsy were censored at their last available PSA follow-up (p. 5). Exclusion of patients with <12 months of follow-up and the potential for attrition bias are described and discussed (pp. 10–12). |
|  |  | (*e*) Describe any sensitivity analyses | N/A |  |
| Results | | | | |
| Participants | 13* | (a) Report numbers of individuals at each stage of study—eg numbers potentially eligible, examined for eligibility, confirmed eligible, included in the study, completing follow-up, and analysed | 4, 6–7 | Numbers at each stage are reported in Section 2.1, Section 3.1, and Figure 1: 582 enrolled, 169 negative mpMRI, 138 biopsied, 8 baseline csPCa, 31 short follow-up, and 99 analysed. |
|  |  | (b) Give reasons for non-participation at each stage | 4, 6 | “Patients with <12 months of follow-up were excluded”; 3 deaths noted; reasons for loss to follow-up not consistently recorded. |
|  |  | (c) Consider use of a flow diagram | 7 | Figure 1. Study design and participant flowchart. |
| Descriptive data | 14* | (a) Give characteristics of study participants (eg demographic, clinical, social) and information on exposures and potential confounders | 6-8 | Participant characteristics and clinical variables are reported in the Results text and Table 1; baseline characteristics of the excluded short-follow-up group are reported in Supplementary Table S2 and referenced on p. 6. |
|  |  | (b) Indicate number of participants with missing data for each variable of interest | N/A |  |
|  |  | (c) *Cohort study*—Summarise follow-up time (eg, average and total amount) | 2, 7 | Mean follow-up was 61.8 ± 22.1 months, reported in the Abstract, Results text, and Table 1. |
| Outcome data | 15* | *Cohort study*—Report numbers of outcome events or summary measures over time | 2, 8–9 | Four of 99 patients developed csPCa; case-level outcomes are reported in Table 2, and csPCa-free survival was 100% at 24 months and 97.3% at 60 months (95% CI 93.7–100%). |
|  |  | *Case-control study—*Report numbers in each exposure category, or summary measures of exposure | N/A |  |
|  |  | *Cross-sectional study—*Report numbers of outcome events or summary measures | N/A |  |
| Main results | 16 | (*a*) Give unadjusted estimates and, if applicable, confounder-adjusted estimates and their precision (eg, 95% confidence interval). Make clear which confounders were adjusted for and why they were included | 2, 6, 8–9 | The absolute event proportion was 4/99 (4.0%), and Kaplan–Meier csPCa-free survival was 97.3% at 60 months (95% CI 93.7–100%). Table 1 is descriptive only; no adjusted estimates were produced because only four events occurred. |
|  |  | (*b*) Report category boundaries when continuous variables were categorized | 4–5, 7–8 | Category boundaries are reported for negative mpMRI (PI-RADS ≤2), minimum follow-up (≥12 months), and <2 versus ≥2 biopsy procedures and mpMRI scans. |
|  |  | (*c*) If relevant, consider translating estimates of relative risk into absolute risk for a meaningful time period | 2, 9 | The absolute 60-month estimate is reported: csPCa-free survival 97.3% (95% CI 93.7–100%). |

Continued on next page

| Other analyses | 17 | Report other analyses done—eg analyses of subgroups and interactions, and sensitivity analyses | 8–9 | Other reported analyses comprise descriptive case-level analysis of the four csPCa diagnoses (Table 2) and Kaplan–Meier csPCa-free survival. No subgroup, interaction, or sensitivity analyses were performed. |
| --- | --- | --- | --- | --- |
| Discussion | | | | |
| Key results | 18 | Summarise key results with reference to study objectives | 10–11 | The Discussion summarises the four csPCa events among 99 patients, the mean follow-up, the selective PSA-triggered pathway, and the 60-month csPCa-free survival estimate. |
| Limitations | 19 | Discuss limitations of the study, taking into account sources of potential bias or imprecision. Discuss both direction and magnitude of any potential bias | 10–12 | Selection and attrition bias are discussed on p. 10. The formal limitations paragraph on pp. 11–12 addresses small sample size, four events, limited generalisability, verification bias, possible underestimation, unavailable outcomes for short-follow-up exclusions, and exclusion of men without baseline SBx. |
| Interpretation | 20 | Give a cautious overall interpretation of results considering objectives, limitations, multiplicity of analyses, results from similar studies, and other relevant evidence | 10–12 | The Discussion interprets the findings cautiously in light of selective repeat investigation, possible underestimation, comparisons with prior studies, few events, and the need for protocol-driven prospective validation. |
| Generalisability | 21 | Discuss the generalisability (external validity) of the study results | 11–12 | Generalisability is addressed through the statement that the small cohort and four events limit generalisability, the selected double-negative cohort may not represent all men with negative mpMRI, and larger prospective studies are required. |
| Other information | |  | | |
| Funding | 22 | Give the source of funding and the role of the funders for the present study and, if applicable, for the original study on which the present article is based | 13 | “This research was conducted as part of a PhD scholarship funded by Taif University, Saudi Arabia. The funder had no role in study design, data collection, analysis, interpretation, manuscript preparation, or the decision to submit for publication.” |

*Give information separately for cases and controls in case-control studies and, if applicable, for exposed and unexposed groups in cohort and cross-sectional studies.

**Note:** An Explanation and Elaboration article discusses each checklist item and gives methodological background and published examples of transparent reporting. The STROBE checklist is best used in conjunction with this article (freely available on the Web sites of PLoS Medicine at http://www.plosmedicine.org/, Annals of Internal Medicine at http://www.annals.org/, and Epidemiology at http://www.epidem.com/). Information on the STROBE Initiative is available at www.strobe-statement.org.
